# Endomyocardial involvement in asymptomatic sub-Saharan immigrants with helminth-related eosinophilia

**DOI:** 10.1371/journal.pntd.0005403

**Published:** 2017-02-24

**Authors:** Cristina Carranza-Rodríguez, Daniel San-Román-Sánchez, Héctor Marrero-Santiago, Michele Hernández-Cabrera, Carlos Gil-Guillén, Elena Pisos-Álamo, Nieves Jaén-Sánchez, José-Luis Pérez-Arellano

**Affiliations:** 1 Department of Medical and Surgery Sciences, University of Las Palmas de Gran Canaria, Las Palmas de Gran Canaria, Spain; 2 Infectious Diseases and Tropical Medicine Division, Complejo Hospitalario Universitario Insular Materno Infantil, Las Palmas de Gran Canaria, Spain; 3 Cardiology Division, Complejo Hospitalario Universitario Insular Materno Infantil, Las Palmas de Gran Canaria, Spain; National Institutes of Health, UNITED STATES

## Abstract

**Background:**

Among immigrants of sub-Saharan origin, parasitic infection is the leading cause of eosinophilia, which is generally interpreted as a defense mechanism. A side effect of the inflammatory mediators released by eosinophils is damage to host organs, especially the heart. The main objectives of this study were to i) assess cardiac involvement in asymptomatic sub-Saharan immigrants with eosinophilia, ii) relate the presence of lesions with the degree of eosinophilia, and iii) study the relationship between cardiac involvement and the type of causative parasite.

**Methodology/Principle findings:**

In total, the study included 50 black immigrants (37 patients and 13 controls) from sub-Saharan Africa. In all subjects, heart structure and function were evaluated in a blinded manner using Sonos 5500 echocardiographic equipment. The findings were classified and described according to established criteria. The diagnostic criteria for helminthosis were those reported in the literature. Serum eosinophil-derived neurotoxin levels were measured using enzyme-linked immunosorbent assay. A significant association was found between the presence of eosinophilia and structural alterations (mitral valve thickening). However, the lack of an association between the degree of eosinophilia and heart valve disease and the absence of valve involvement in some patients with eosinophilia suggest the role of other factors in the appearance of endocardial lesions. There was also no association between the type of helminth and valve involvement.

**Conclusions:**

We, therefore, suggest that transthoracic echocardiography be performed in every sub-Saharan individual with eosinophilia in order to rule out early heart valve lesions.

## Author summary

Endomyocardial fibrosis is characterized by fibrosis of the apical endocardium of the right ventricle, left ventricle, or both. Epidemiological studies of endomyocardial fibrosis indicate a predominance in tropical regions, with young people and men being affected predominantly. Little is known about the natural history and pathogenic factors of this condition. One of the most important factors is the presence of eosinophilia, which is mainly related to helminth infections. The aim of the study was to evaluate cardiac involvement in patients with absolute eosinophilia. We performed an echocardiographic study in asymptomatic sub-Saharan immigrants with eosinophilia and compared them with a group of control patients from the same region and of the same age. Our results suggest that eosinophilia associated with helminth infection (regardless of the causative microorganism) contributes to the asymptomatic phases of endomyocardial involvement. Treatment of these infections is simple, effective, and economical. Therefore, we suggest that all immigrant patients with eosinophilia undergo etiologic study and causal treatment as well as transthoracic echocardiography in order to rule out early damage.

## Introduction

Eosinophilia is a common finding among immigrants, especially those of sub-Saharan origin. In this population, the main cause of eosinophilia is parasitic infection, specifically by helminths [1]. There now are sufficient data that demonstrate that eosinophils, through their proinflammatory mediators such as eosinophil-derived neurotoxin (EDN) and eosinophil cationic protein, play an important role in the control of the larval phase of these parasitic diseases [2,3]. Thus, helminth-induced eosinophilia may be seen as a defense mechanism.

A side effect of the inflammatory mediators released by eosinophils is lesions in host organs, with cardiac involvement being especially characteristic [4,5]. Injury to the heart structures associated with eosinophilia may take two clinical forms: acute and subacute or chronic. The acute forms follow two main patterns: acute necrotizing eosinophilic myocarditis and hypersensitivity myocarditis [5,6]. The subacute or chronic forms also have two polar forms: Davies’ endomyocardial fibrosis, found in tropical areas, and Loeffler’s fibroblastic endocarditis, which occurs in temperate regions [4,7]. Regardless of the clinical form, cardiac involvement in parasitic-induced eosinophilia has been documented in epidemiological studies as well as in experimental clinical and animal models [8–10].

Therefore, the objectives of this study were to i) evaluate cardiac involvement in asymptomatic sub-Saharan immigrants with eosinophilia; ii) relate the presence of lesions with the degree of eosinophilia; and iii) study the relationship between cardiac involvement and the type of causative parasite.

## Methods

### Ethics statement

This study was approved by the Ethics Committee of the Complejo Hospitalario Insular-Materno Infantil (Las Palmas de Gran Canaria, Spain). Written informed consent was obtained from the participants in their local languages. The reasons for the collection and storage of blood samples and echocardiography were clearly described in the consent form.

### Study population

In total, 52 black immigrants from sub-Saharan Africa, namely West Africa, were included in the study. Their mean age was 25 years and the male/female ratio was 47/5. Two patients were excluded, owing to rheumatic mitral valve disease in one case and mitral valve prolapse associated with mild mitral valve insufficiency in the other. Therefore, the final study group consisted of 37 patients and 13 controls.

The subjects, who met following inclusion criteria, were recruited consecutively during external consultations at the Unit of Infectious Diseases and Tropical Medicine: i) absence of clinical manifestations of heart disease, ii) presence of absolute eosinophilia (>450/μL), and iii) explicit consent to participate in the study. Eosinophilia was classified as mild (450–999/μL), moderate (1,000–1,499/μL), or severe (>1,500/μL).

The control group consisted of asymptomatic subjects from the same geographic region in whom the presence of eosinophilia and/or parasitosis were ruled out by means of the indicated tests, who provided consent to participate in the study. Table 1 summarizes the demographic characteristics of each group.

**Table 1:** Demographic Data and General Characteristics.

	Study Group (Eosinophils >450 cells/μL)	Control Group (Eosinophils <450 cells/μL)	p-Value
**Patients** (n)	37	13	-
**Age, years** (Mean; SD)	25 (6)	26 (3)	0.54*
**Sex** (Male/Female)	34/3	13/0	0.56**
**Country** (Alphabetical order)			
Cameroon	5	1	
Ghana	7	2	
Guinea	2	0	
Guinea-Bissau	1	1	
Equatorial Guinea	2	0	
Mali	5	4	
Mauritania	0	1	
Nigeria	2	0	
The Gambia	1	0	
Sierra Leone	12	3	
Togo	0	1	0.36***
**Eosinophilia** (Mean; SD)	932.1 ± 443.8	218.3 ± 108.9	-
Mild (450–999/μL),	26 (70.2%)	-	
Moderate (1000–1499/μL)	7 (19%)	-	
Severe (>1500/μL)	4 (10.8%)	-	
**Parasitic infection**			
Schistosomal species	8 (21.6%)	-	
Species causing filariasis	13 (35.1%)	-	
Geohelminths	7 (18.9%)	-	
Co-parasitized	4 (10.8%)	-	

*Student’s t-test

**Fisher’s exact test

***Chi-square test

### Parasitological techniques

All parasitological studies (direct and serological) were performed in the same specialized laboratory using the investigation strategy suggested by Whetham for African patients, with some modifications [11].

The direct parasitological tests used in this study were i) three examinations of stool samples for ova and parasites using the Kato-Katz and Ritchie techniques and specific tests for *Strongyloides stercoralis* (Baermann concentration test and agar culture method) [12], ii) microscopy of a terminal urine sample, and iii) Knott’s test for the detection of microfilaremia. The Immune Chromatographic Test (ICT Filariasis Binax, Portland, Maine) was used for the detection of *Wuchereria bancrofti* antigens, with skin snips and the Mazzotti test used in selected cases.

The serological assays were performed using enzyme-linked immunosorbent assay (ELISA). Crude extracts of adult *Dirofilaria immitis* worm (AWA Di) [13], *Schistosoma bovis* adult worm antigens (AWA Sb) [14], *F*. *hepatica* excretory/secretory antigens (E/S Fh) [15], and *Trichinella spiralis* L1 worm antigens (L1 Ts) [16] were used, respectively, for the detection of filariasis, schistosomiasis, fascioliasis, and trichinellosis. Polystyrene microtiter plates (Costar) were coated with 100 μL of antigens per well at a previously determined protein concentration in carbonate buffer (pH 9.6). Serum at a dilution of 1:100 was added to the wells and incubated for 1 h at 37°C. Horseradish peroxidase goat anti-human immunoglobulin G (Sigma, St. Louis, MO, USA) was added at different dilutions. Washes were performed three times with 200 μL of phosphate buffered saline-Tween 20 per well. After incubation for 1 h at 37°C, the substrate solution *(orth*o-phenylenediamine plus H_2_O_2_) was added and the reaction was stopped with 3N H_2_SO_4_. The individual conditions of the assays and the sensitivity and specificity of the tests are described in our previous publication [1].

### Echocardiographic studies

In order to assess the degree and type of cardiac involvement in the study and control group subjects, two-dimensional, M-mode, and Doppler evaluations were performed using the same echocardiographic equipment (Sonos 5500; Philips Medical Systems, Andover, MA, USA). The tracings were recorded for playback later on and blinded analysis by a single investigator.

The following determinations were made for each patient: i) The dimensions of the left and right ventricular chambers and the left atrium were measured using the M-mode technique in the parasternal long and short axis planes. ii) The morphological characteristics of the heart were analyzed using two-dimensional echocardiography (2D echo), with optimization of the image by adjusting the frequency and parameters of gain, compression, and image processing. Standard parasternal long and short axes as well as four- and two-chamber apical and subcostal planes were used. A high-resolution zoom magnification system was applied to specific areas, including the ventricular apical zones and valvular planes, for better visualization and quantification of possible alterations. iii) The left ventricular ejection fraction was analyzed using 2D echo, using the Simpson biplane technique. iv) The functional parameters of ventricular filling were analyzed using pulsed-wave Doppler ultrasound of transmitral and transtricuspid flow during mid-expiratory apnea, with calculation of the initial peak velocities (E in cm/s), slope deceleration (TD in milliseconds), speed atrial contraction (A in cm/s), and E/A ratio. If possible, the pulmonary venous flow (apical four-chamber plane) with calculation of the peak systolic (S in cm/s) and diastolic (D in cm/s) velocities and S/D ratio, and peak velocity (cm/s) and duration (in milliseconds) of the retrograde atrial wave (Ar) were measured. In all cases, measurements of a minimum of four consecutive heartbeats were analyzed. v) The presence of valve regurgitation was evaluated using color-coded Doppler ultrasound in the parasternal planes, long and short axes, and apical four and two chambers. vi) In cases with tricuspid insufficiency, the pulmonary systolic pressure was calculated using the Bernoulli equation: 4V^2^+10 (in mm Hg) (where V represents the maximum velocity).

The findings were classified into two groups according to the following descriptions from the literature on cardiac involvement in the presence of eosinophilia [17,18]:

i) **Classic findings:** Endocardial thickening, described as thickening of the wall, with refringent and additional echogenic layers in the endocardial zone; thrombus in the left ventricle, characterized by an echodense mass at the left cardiac apex, causing obliteration or limitation of the blood flow during filling; apical thrombus in the right ventricle, with the same characteristics as on the left side; affectation (thickening) of the mitral valve leaflets, especially the posterior leaflet, determined by thickness values greater than 3 mm; and tricuspid insufficiency, defined as the detection of signals of tricuspid valve insufficiency on color-coded Doppler with a semiquantitative assessment of its severity

ii) **Non-classic findings:** Left ventricular hypertrophy, defined according to the criteria described by Casale et al. [19] using measurements of interventricular septum thickness (IST) and posterior wall thickness (PWT) (the degree of left ventricular hypertrophy was classified as mild [IST = 12–13 mm; PWT = 11–12 mm], moderate [IST = 13–14 mm; PWT = 12–13 mm], or severe [IST = 14–15 mm; PWT = 13–14 mm]); left ventricular dilatation, defined as ventricular transverse diastolic diameter >56 mm; pericardial effusion, the presence of echo-free spaces of more than 5 mm in the left ventricular posterior wall; hyperdynamic left ventricle, characterized by a ventricular ejection fraction equal to or greater than 70%; and severe left ventricular diastolic dysfunction, defined as an E/A ratio of mitral transvalvular flow >2 and/or TD <130 milliseconds.

### Determination of serum EDN levels

Serum EDN levels were measured by sandwich ELISA using a commercial kit (EDN ELISA kit ref 7630, MBL International Corporation, USA) following the manufacturer instructions. The limit of detection of the technique is 0.62 ng/mL [20]. The normal values are ≤66.6 ng/mL [21].

### Statistical methods

Qualitative variables were expressed as frequency distributions. Quantitative variables with normal distributions were expressed as the mean and standard deviation (SD). The associations between qualitative variables were analyzed using the chi-square or Fisher’s exact test, as appropriate. Normally distributed variables were compared using the Student’s t-test. Data distribution as compared with theoretical models was verified, and the assumption of the homogeneity of variance was tested in all cases. Analyses were performed using GraphPad InStat. Differences were considered significant if p values were less than 0.05.

## Results

In the study group, the degree of eosinophilia was mild in 26 (70%), moderate in seven (22%), and severe in four (8%) patients. The etiological diagnosis was filariasis in 13 cases, schistosomiasis in eight cases, coexistence of filariasis and schistosomiasis in four cases, a geohelmintic disease in seven cases, and indeterminable in five people. The relationship between the degree of eosinophilia and the causal diagnosis is shown in Table 2.

**Table 2:** Types of Eosinophilia and Final Diagnoses.

	n	Eosinophil Counts (Mean; SD)	Final Diagnosis
Mild (450–999/μL)	26	721 (444)	Filarial diseases (9) Schistosomal diseases (8) Geohelmintic diseases (5) Undetected (4)
Moderate (1,000–1,499/μL)	7	1,119 (63)	Filarial diseases (2) Filarial/Schistosomal diseases (2) Geohelmintic diseases (2) Undetected (1)
Severe (>1,500/μL)	4	1,975 (515)	Filarial diseases (2) Filarial/Schistosomal diseases (2)

**Fig 1:**
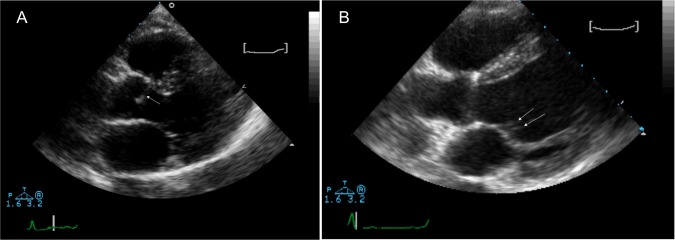
Characteristic Echocardiographic Findings. (A) Two-dimensional echocardiography image: parasternal long axis showing a thickening of the aortic sigmoid (arrows) without altering its function. (B) Parasternal long axis view of the left ventricle, showing slight thickening of the mitral leaflets (arrows), the most frequent finding in this study.

All the subjects had an adequate echocardiographic image and were in sinus rhythm, with an average heart rate of 69 beats/minute (SD: 11). All subjects had normal blood pressure, with a mean value of 125/75 mm Hg. Tables 3 and 4 show the qualitative and quantitative echocardiographic results as well as their statistical analyses. As can be observed, a significant association was found only between thickening of the mitral valve and the presence of eosinophilia, with the thickness of the mitral valve being significantly greater in cases with eosinophilia than in those without eosinophilia. Two representative images are shown in Fig 1.

**Table 3:** Qualitative Echocardiographic Findings.

	Study Group (Eosinophils >450 cells/μL)	Control Group (Eosinophils <450 cells/μL)	p-Value
**Classic findings**			
Endocardial thickening	2/37	0/13	1.0*
Left ventricle apical thrombus	1/37	0/13	1.0*
Right ventricle apical thrombus	0/37	0/13	1,0*
Posterior mitral leaflet thickening	18/37	0/13	0.001*
Tricuspid valve thickening	7/37	0/13	0.16*
Tricuspid insufficiency	15/37	4/13	0.08**
Aortic valve thickening	2/37	0/13	1.0*
**Non-classic findings**			
Left ventricular hypertrophy	3/37	0/13	1.0*
Dilated left ventricle	0/37	0/13	1.0*
Hyperdynamic left ventricle	1/37	0/13	1.0*
Pericardial effusion	0/37	0/13	1.0*
Severe left ventricular diastolic dysfunction	0/37	0/13	1.0*

* Fisher´s exact test

** Chi-square test

**Table 4:** Quantitative Echocardiographic Findings (Mean and Standard Deviation).

	Study Group (Eosinophils >450 cells/μL)	Control Group (Eosinophils <450 cells/μL)	p-Value
Posterior mitral leaflet thickening	2.98 (0.67) mm	2.47 (0.39) mm	**0.002**
Tricuspid valve thickening	2.37 (0.69) mm	2.04 (0.49) mm	0.079
Aortic valve thickening	1.89 (0.56) mm	1.75 (0.31) mm	0.32
Left ventricular ejection fraction	56.67 (4.91) %	56.38 (3.93) %	0.83
DTI (E/A) of the mitral annulus	2.02 (0.55)	2.12 (0.73)	0.64
Pulmonary venous flow (S/D)	1.13 (0.31)	1.11 (0.38)	0.86
Mitral valve E/A ratio	1.72 (0.59)	1.76 (0.52)	0.81
DTI (E/A) of the basal septum	1.57 (0.38)	1.46 (0.32)	0.38
Tricuspid valve E/A ratio	1.92 (0.48)	1.80 (0.43)	0.45
DTI (E/A) of the middle lateral wall	1.84 (0.46)	1.90 (0.28)	0.59
DTI (E/A) of the middle septum	1.64 (0.43)	1.60 (0.31)	0.77
IVRT	74.61 (13.63) ms	74.07 (11.95) ms	0.89
Color M-mode flow propagation velocity	56.32 (18.06) cm/s	50.11(12.12) cm/s	0.19

DTI, Doppler tissue imaging; IVRT, isovolumetric relaxation time.

The serum concentration of EDN was determined in 29 patients with eosinophilia, 28 of whom had high values (>66.6 ng/mL). However, there were no significant differences (p = 0.46) in the serum concentration of this protein between patients with valve involvement (115.5 ng/mL, SD: 36.6) and those without it (129.1 ng/mL, SD: 53.8). On the other hand, there was no statistically significant association between the number of eosinophils and serum levels of EDN (p = 0.18).

Further, there was no statistical association between the degree of eosinophilia and the presence of valve involvement, or between the type of helminth responsible for eosinophilia and valve involvement, as shown in Table 5.

**Table 5:** The Association Between Valve Involvement and the Type of Helminth.

Valve Involvement	*Schistosoma* spp.	Species causing filariasis	Geohelminth	Total
Yes	8	6	1	15
No	3	9	6	18
Total	11	15	7	33

Fisher’s exact test (Freeman-Halton extension), p = 0.07

## Discussion

Endomyocardial fibrosis (EMF) is a syndrome characterized by fibrosis of the apical endocardium of the right ventricle, left ventricle, or both. The clinical manifestations are mainly related to the restriction of ventricular filling, which may trigger cardiac failure.

Epidemiological studies of EMF indicate a predominance in tropical regions (15° around the equator), with young people (with a bimodal peak at 10 and 30 years of age) and men being affected predominantly. In this sense, our series is similar to that of patients with EMF in terms of age and sex, although our series included a greater number of people from West Africa. In some series, EMF data were available for up to 19.8% of the population studied by echocardiography, and only 22.7% of these had symptoms [22]. These data suggest that EMF clinical series mainly include patients in advanced stages of disease.

The pathogenesis of EMF is not fully understood, although several types of factors have been identified. One of the most important factors is the presence of eosinophilia related to helminth infections or other causes (e.g., hypereosinophilic syndrome, Churg-Strauss disease, reactions to drugs, or neoplasia) [23–25]. The association between the presence of eosinophilia and EMF varies greatly depending on the geographical region (e.g., highest in Uganda [26], low in India [27]). The other factors involved are nutritional (low protein diet, cassava consumption) [7], environmental (increased cerium) [27], genetic [22], and immunological [28].

Therefore, the first objective of our study was to compare the echocardiographic findings of asymptomatic sub-Saharan immigrants from West Africa with eosinophilia with a group of controls from the same region and of equivalent age. Our results indicate that there is a significant association between the presence of eosinophilia and structural alterations, in the absence of evident clinical manifestations. Thus, mitral valve thickening suggests early involvement, independent of the racial characteristics and other factors controlled for in the study (e.g., age or sex). There were significant differences in the structure of the leaflets of the mitral valve, with a significantly greater thickness in cases with eosinophilia. These data may have clinical relevance since the predominant involvement of the mitral valve in patients with EMF and eosinophilia has been demonstrated in other studies as well [29–33]. On the other hand, it is interesting to note that eosinophilia and/or the presence of parasitosis in this context do not give rise to alterations in left ventricular contractile function (expressed as a percentage of the ejection fraction) or alter the global or regional diastolic function of the left or right side of the heart.

Although eosinophilia plays an important role in the pathogenesis of EMF, there is no direct relationship between the two. Thus, in our series, we observed on the one hand that a significant percentage of patients with eosinophilia did not present any data suggesting cardiac involvement; on the other hand, there was no association between the degree of eosinophilia and cardiac valve involvement. One possible explanation for these findings is that not only is an increase in eosinophils required for the development of EMF but that these are also activated and release inflammatory mediators [34]. However, our data do not support this interpretation since in nearly all patients with eosinophilia, there was an increase in serum EDN levels. In summary, eosinophilia plays an important role in the development of EMF, although other factors (e.g., genetics, nutritional factors, duration of helminthic infection) can contribute to its appearance [35].

In our series, the cause of eosinophilia was identified by direct or indirect techniques in 86% of cases. In all cases, it was a helminthic disease, mainly filariasis, schistosomiasis, or both. Cardiac involvement in helminths has three main patterns: i) myocarditis due to direct or indirect myocardial involvement in *Trichinella* spp. infection [36]; ii) acute endocarditis in *Toxocara* spp. infection [37,38], and iii) endomyocardial fibrosis. Infection with several helminths is associated with EMF, although infection by different species of *Filarias* is the most frequently described association [39–41]. However, in our series, there was no statistical association between valve involvement and the type of parasite. In fact, we observed this association with *Schistosoma* spp. infection, which is rarely indicated in the literature [42–44].

These data suggest that eosinophilia related to helminth infection (regardless of the causative microorganism) contributes to the asymptomatic phases of endomyocardial lesions. The treatment of these infections is simple, effective, and cheap. Therefore, we suggest that an etiological study and causal treatment, as well as transthoracic echocardiography, be performed in all immigrant patients with eosinophilia in order to rule out early lesions.
